# Risk Stratification, Exercise Prescription, and Sport Participation in Individuals with Aortic Valve Disease

**DOI:** 10.3390/jcdd13100497

**Published:** 2026-10-03

**Authors:** Francesco Perone, Edoardo Conte, Stefano Palermi, Geza Halasz, Marco Ambrosetti, Luigi Sciarra, Giacomo Frati, Mariangela Peruzzi, Elena Cavarretta, Annachiara Pingitore

**Affiliations:** 1 Cardiac Rehabilitation Unit, Rehabilitation Clinic “Villa delle Magnolie”, 81020 Castel Morrone, Italy; 2 Department of Clinical Cardiology and Cardiovascular Imaging, IRCCS Galeazzi-Sant’Ambrogio Hospital, Via Cristina Belgioioso 173, 20157 Milan, Italy; edoardo.conte86@gmail.com; 3 Department of Medicine and Surgery, UniCamillus-Saint Camillus International University of Health Sciences, 00131 Rome, Italy; stefano.palermi@unicamillus.org; 4 Division of Cardiology, Cardio-Thoracic and Vascular Department, Azienda Ospedaliera San Camillo Forlanini, Cir.ne Gianicolense 87, 00152 Rome, Italy; geza.halasz@gmail.com (G.H.); annachiara.pingitore@gmail.com (A.P.); 5 Department of Cardiac Rehabilitation, Istituti Clinici Scientifici Maugeri IRCCS, 27100 Pavia, Italy; marco.ambrosetti@icsmaugeri.it; 6 Department of Life, Health and Environmental Sciences, University of L’Aquila, 67100 L’Aquila, Italy; luigi.sciarra@univaq.it; 7 Department of Medical-Surgical Sciences and Biotechnologies, Sapienza University of Rome, Corso Della Repubblica 79, 04100 Latina, Italy; giacomo.frati@uniroma1.it (G.F.); elena.cavarretta@uniroma1.it (E.C.); 8 IRCCS Neuromed, 86077 Pozzilli, Italy; 9 Department of Medical and Cardiovascular Sciences, Sapienza University of Rome, 00185 Rome, Italy; mariangela.peruzzi@uniroma1.it; 10 IRCCS Maria Cecilia Hospital, GVM Care & Research, 48033 Cotignola, Italy; 11 Heart Failure, Transplant and Mechanical Assist Device Unit, Bambino Gesù Children’s Hospital IRCCS, 00165 Rome, Italy

**Keywords:** bicuspid aortic valve, aortic stenosis, aortic regurgitation, risk stratification, exercise prescription, pre-participation screening, athletes, sport participation, transcatheter aortic valve implantation, surgical aortic valve replacement

## Abstract

Aortic valve disease (AVD), including bicuspid aortic valve (BAV) and aortic regurgitation (AR), presents a complex challenge in sports cardiology. Balancing the cardiovascular benefits of physical activity with the hemodynamic risks associated with valvular dysfunction and aortopathy requires precise risk stratification. The present review comprehensively analyses the contemporary criteria for exercise prescription and competitive sports participation in individuals with AVD, highlighting also the distinctions between the European guidelines (European Society of Cardiology—ESC), the Italian Sports Cardiology Guidelines for competitive sport practice (COCIS 2023) and the recommendations for exercise prescription in cardiovascular diseases promoted by the Italian Sports Cardiology Society (SIC Sport) and the Italian Federation for Sports Medicine (FMSI). Relevant differences with the 2025 AHA/ACC recommendations are also highlighted where they may influence sports eligibility. Eligibility hinges primarily on the degree of valvular defect, left ventricular ejection fraction (LVEF), and aortic dimensions. Furthermore, post-operative sports participation is highly contingent on the intervention type, post-operative results and follow-up. Ultimately, safe exercise prescription relies on meticulous clinical surveillance, advanced imaging, maximal stress testing, and a shared decision-making process to maximize cardiovascular health while mitigating the risk of acute adverse events.

## 1. Introduction

Aortic valve disease (AVD), encompassing a spectrum of disorders affecting the aortic valve, is a significant cardiovascular condition impacting millions globally. It primarily includes aortic stenosis and aortic regurgitation, each presenting unique challenges in clinical management [1,2]. The prevalence of AVD increases with age, making it a growing concern in an aging population [3]. The disease’s progression can lead to debilitating symptoms and increased mortality risk, necessitating timely diagnosis and intervention. The management of AVD has traditionally focused on medical and surgical treatments. However, the role of lifestyle modifications, particularly exercise and sport participation, has gained attention for its potential to improve quality of life and cardiovascular outcomes in several cardiovascular disease populations, including patients with AVD [4]. Risk stratification in AVD is pivotal for tailoring treatment and monitoring strategies. It involves assessing the severity of the disease, the presence of symptoms, and the patient’s overall cardiovascular risk [5]. This process guides clinicians in decision-making, from conservative management to considering valve replacement or repair. Competitive sport participation for AVD patients is a nuanced area. While physical activity is beneficial for cardiovascular health, the type, intensity, and duration of exercise must be carefully considered to avoid exacerbating the condition [6,7]. Current guidelines offer insights but also underscore the need for individualized exercise regimens based on patient-specific assessments. Sport participation, once considered risky for AVD patients, is now being re-evaluated. Emerging evidence suggests that, with proper risk stratification and monitoring, certain sports might be safe and beneficial for these individuals. Even in patients who underwent surgical intervention, in selected cases, after a careful case-by-case evaluation, even competitive sport can be resumed (Table 1).

This review seeks to provide a comprehensive overview of risk stratification, exercise prescription, and sport participation in individuals with AVD, emphasizing the need for a balanced and patient-centric approach in managing this complex condition. Given the primarily European perspective of this review, the 2025 AHA/ACC recommendations are specifically discussed where they differ meaningfully from European guidance, thereby highlighting clinically relevant differences in sports eligibility [8].

## 2. Bicuspid Aortic Valve

### 2.1. Diagnosis and Risk Stratification

Bicuspid aortic valve (BAV) is the most common congenital cardiac malformation and occurs in approximately 0.5–2% of the population, with a higher prevalence in males [9,10,11]. BAV encompasses a heterogeneous morphological spectrum—most commonly characterized by the fusion of two aortic cusps—which inherently predisposes individuals to progressive valvular dysfunction and aortopathy. The International Consensus Classification recognizes three overarching valve phenotypes—fused type, two-sinus type, and partial-fusion (forme fruste)—and recommends reporting raphe presence and commissural symmetry, since these traits relate to the valve rate of degeneration and flow-mediated wall stress [12].

Transthoracic echocardiography (TTE) is the first-line imaging modality for diagnosis, grading of aortic stenosis (AS) or regurgitation (AR), and measurement of aortic dimensions from the annulus to the proximal arch. When the acoustic images are suboptimal or when a precise assessment of root and tubular ascending aorta is required, transesophageal echocardiography (TEE), electrocardiogram-gated cardiac magnetic resonance (CMR), or computed tomography angiography (CTA) should be used. Serial aortic imaging is mandatory to determine eligibility for competitive sports and exercise prescription [4,13]. A recent multicenter international study enrolled 186 competitive athletes with BAV (84% males; age 30 ± 11 years), 193 competitive athletes with tricuspid aortic valve (TAV) and 125 non-athletes with BAV and demonstrated that athletes with BAV have larger aortic diameters and valve impairment, when compared with athletes with TAV, but no significant differences were found when comparing athletes vs. non-athletes, both with BAV [14,15].

Risk stratification integrates: (1) valvular lesion severity and symptoms; (2) aortic dimensions (absolute values and indexed/z-scores) and rate of enlargement; (3) associated congenital or syndromic conditions (e.g., coarctation, Turner syndrome, heritable thoracic aortic disease); (4) family history of aortic dissection or sudden cardiac death; and (5) functional capacity and exercise blood pressure response, preferably assessed by cardiopulmonary exercise testing (CPET). Twenty-four-hour ambulatory blood pressure monitoring (ABPM) may complement office and exercise blood pressure assessment in selected athletes, particularly in those with borderline blood pressure or an exaggerated exercise blood pressure response [4]. This may be especially relevant in patients with concomitant aortopathy, in whom optimal blood pressure control is an important component of risk reduction [10]. Lifetime morbidity is high driven by progressive valve disease, aortic dilatation, and infective endocarditis, with global survival comparable to the general population in ‘typical’ BAV, while ‘complex’ phenotypes carry excess mortality [16].

### 2.2. Aortopathy Associated with BAV

BAV aortopathy comprises dilatation of the aortic root and/or ascending aorta with histopathological features of tunica media degeneration and extracellular matrix remodelling. Its pathogenesis reflects an interplay between inherited defects (e.g., NOTCH1, ACTA2 and TGF-β pathway disorders) and abnormal hemodynamic shear stress from asymmetric valve orifice jets [17,18,19]. Distinct phenotypes include: (1) root-predominant dilatation (15–20%—often syndromic and associated with higher dissection risk), (2) tubular ascending dilatation (70–75%—most common in isolated BAV), and (3) extended forms involving the arch (5–10%).

Typical growth rates are modest (≈0.2–0.5 mm/year), but faster progression may occur with root phenotype, male sex, hypertension, or when baseline diameter is larger.

According to the latest aortic disease ESC guidelines, elective surgery for bicuspid aortopathy is recommended at ≥55 mm of aortic diameter (Class I—Level B), but when the surgical risk is low the replacement of the ascending phenotype might be considered at >52 mm (Class IIa—Level B) or at >50 mm (Class IIa—Level C) when additional risk factors are present (age < 50 years, patient height between 1.50 and 1.69 m, ascending aortic length ≥ 11 cm, aortic diameter growth rate ≥ 3 mm per year, family history of acute aortic syndrome, aortic coarctation, resistant hypertension, desire for pregnancy). A lower threshold of ≥50 mm applies in root-phenotype BAV (Class I—Level B). When aortic valve surgery is planned, concomitant aortic replacement should be considered from ≥45 mm (Class IIa—Level C). Moreover, the cross-sectional aortic area/height ratio ≥ 10 cm^2^/m may support intervention in borderline diameters after Heart Team discussion [5,11]. Absolute aortic diameters should not be interpreted in isolation and should be contextualized according to body size, using indexed measurements or Z-scores, when appropriate [4,10]. This is particularly relevant in very tall or small individuals, in paediatric athletes, in whom unindexed aortic diameters alone may misrepresent the degree of aortic dilatation. Nevertheless, absolute dimensions remain central to risk stratification and should be integrated with indexed measurements, serial assessment, and aortic growth rate [4,10]. Notably, recent AHA/ACC recommendations similarly advocate consideration of body size but retain absolute aortic size as the primary metric because of the stronger outcome data supporting this approach [8].

From an exercise perspective, aortic size and stability are pivotal determinants. Evidence suggests that mild–moderate dynamic aerobic training does not accelerate aortic enlargement in heritable aortopathies [4,20], whereas activities provoking abrupt systolic blood pressure surges (e.g., heavy isometrics, maximal strength efforts) may impose disproportionate aortic wall stress. Given the paucity of prospective data in BAV, recommendations remain cautious and individualized [4,20,21].

### 2.3. Exercise Prescription

For the purposes of cardiovascular prevention, rehabilitation, and recreational sports—as distinct from competitive sports clearance—exercise prescription in individuals with BAV should primarily be guided by the presence and extent of aortic dilatation. In case of BAV without aortopathy, exercise recommendations are comparable to those applied to patients with tricuspid aortic valve disease.

When the ascending aorta remains within normal limits (<40 mm), individuals with BAV are considered at low risk, and all types of training are generally permitted, with a preference for aerobic endurance over strength activities. Regular exercise should be encouraged given its well-established cardiovascular benefits, and standard clinical surveillance every 2–3 years is sufficient in this setting [4].

As aortic dimensions exceed normal values, expert consensus panels advocate a progressively more cautious approach. In patients with mild aortic dilatation (40–45 mm), corresponding to a low–intermediate risk profile, participation in structured physical activity remains acceptable but should favor low-to-moderate intensity aerobic exercise, while high- and very high-intensity activities, contact sports, and power or strength training should be avoided. Endurance activities are preferred over interval training, and closer follow-up (every 1–2 years) is recommended.

With moderate aortic dilatation (typically 45–50 mm in BAV), patients fall into an intermediate risk category, and exercise prescription should be restricted to low-intensity activities, including skill-based sports or mixed/endurance disciplines performed at submaximal effort. At this stage, structured exercise should be individualized, blood pressure responses carefully monitored, and follow-up intensified to every 6–12 months.

In the presence of severe aortic dilatation (>50 mm in BAV) or after surgery with residual aortic sequelae, individuals are classified as high risk, and training programs are temporarily contraindicated until definitive treatment is undertaken and clinical stability is confirmed on follow-up imaging [5].

### 2.4. Competitive Sport Participation

According to ESC 2020 guidelines [4], athletes with normally functioning BAV and normal aortic dimensions (<40 mm) may participate in all competitive sports without restriction. Those with mild valvular dysfunction or aortic dilatation up to 45 mm may engage in low-to-moderate dynamic and low-static disciplines (e.g., cycling, swimming, running), provided that blood pressure and rhythm are within normal limits during exercise testing and no aortic enlargement is observed over time. More specifically, in athletes with BAV, an appropriate exercise response should include a progressive rise in systolic blood pressure without an excessive hypertensive response; peak SBP > 220 mmHg in men or >200 mmHg in women is above the 95th percentile in young athletes and warrants further evaluation [4]. Exercise testing should also exclude clinically relevant exercise-induced rhythm disorders, particularly complex or sustained ventricular arrhythmias [4,5]. Competitive sports are explicitly contraindicated when the aortic diameter is ≥45 mm, or in the presence of moderate-to-severe aortic stenosis or regurgitation, progressive dilatation (>3 mm/year), or a history of aortic dissection. In contrast, the COCIS 2023 protocol [12], which guides Italian medical eligibility, applies a more conservative approach for competitive sports (Table 2).

The COCIS [12] classifies BAV in different classes:

- Class I (eligible)—uncomplicated BAV (neither stenosis nor regurgitation and normal aortic dimensions) in normotensive individuals, can engage in all competitive sports, after cardiovascular overall assessment (clinical examination, echocardiography, exercise testing) at baseline and repeated every 6–12 months;
- Class II (conditionally eligible)—BAV with mild valve dysfunction and/or aortic diameter 40–45 mm, allowing only disciplines with low-static and dynamic components (e.g., golf, shooting, bowling), after individualized assessment;
- Class III (not eligible)—BAV with moderate or severe valve disease, aorta ≥45 mm, rapid progression, or syndromic/heritable aortopathy, where participation in competitive sports is contraindicated regardless of training level, with the exception of skill sports in selected individuals with significant aortic stenosis after tailored clinical assessment.

The 2025 AHA/ACC statement, conversely, adopts a less restrictive approach, allowing competitive sport participation through shared decision-making in athletes with BAV and aortic diameters of 40–44 mm after accounting for age, sex, body size, and additional aortic risk factors; even at diameters ≥ 45 mm, although risks are considered likely to outweigh benefits, participation may still be considered in selected athletes after specialist evaluation and with close longitudinal surveillance [8].

## 3. Aortic Stenosis

### 3.1. Pathophysiology

Aortic stenosis (AS) mainly arises from degenerative calcific valve disease in the elderly. It leads to LV outflow obstruction, causing compensatory LV hypertrophy and impaired diastolic function leading to increased myocardial oxygen consumption and potential risk of myocardial ischemia especially during exercise. While LV volume remains normal, an increased myocardial thickness and enlarged left atrial volumes may indicate hemodynamic overload in asymptomatic but severe AS. Symptoms like syncope or presyncope often precede late-stage symptoms of angina or dyspnea, with a higher risk of sudden cardiac death demonstrated among symptomatic patients. Bicuspid aortic valve (BAV) is a congenital heart disease, affecting 1–2% of the population, often leading to AS or aortic regurgitation in the young and increasing the risk of aortopathy. Factors like body size, age, and blood pressure influence aortic root dimensions, and rapid aortic dilatation may indicate the presence of an aortopathy that could be associated with BAV.

### 3.2. Diagnosis and Risk Stratification

Aortic stenosis is often first identified during clinical evaluation through heart auscultation. An intense and diffuse systolic murmur is easily audible in the presence of severe aortic stenosis. Doppler echocardiography is then used to measure the pressure gradient across the aortic valve and determine the valve area. Echocardiography parameters are of utmost importance for diagnostic evaluation and risk stratification (Table 3). More specifically, severe AS is defined by a transvalvular Doppler velocity ≥ 4.0 m/s; a mean gradient ≥ 40 mmHg; and a calculated aortic valve area < 1.0 cm^2^ or an indexed area (recommended in athletes) < 0.6 cm^2^/m^2^. Exercise testing is advised for individuals with mild-to-moderate aortic stenosis to evaluate the presence of symptoms during physical stress, the hemodynamic response, and potential arrhythmias. More specifically, exercise testing should be symptom-limited and, whenever feasible, use a protocol that approximates the cardiovascular demands of the intended sport, avoiding premature termination at arbitrary submaximal workloads [4]. Continuous ECG monitoring and repeated blood pressure measurements should assess functional capacity, symptoms, haemodynamic response, ischaemia, and exercise-induced arrhythmias [1]. CPET provides additional objective assessment of exercise capacity (including peak VO_2_, ventilatory threshold, ventilatory efficiency, oxygen pulse, and chronotropic response) and may be particularly useful when symptoms or functional limitation are uncertain. Testing should be performed under experienced supervision and terminated for limiting symptoms, exercise-induced hypotension, significant arrhythmias, ischaemic changes, or other signs of haemodynamic instability [5].

Stress echocardiography is crucial for those with at least moderate AS, revealing dynamic valvular abnormalities and latent myocardial dysfunction not apparent at rest. Low-flow, low-gradient aortic stenosis with reduced ejection fraction is characterized by a mean gradient less than 40 mmHg, a valve area of 1 cm^2^ or less, and a left ventricular ejection fraction (LVEF) under 50%. Low-dose dobutamine stress echocardiography is used to differentiate between true severe and pseudo-severe aortic stenosis and to assess flow reserve, though its utility in the elderly is based on small registries. In cases where there is low-flow, low-gradient aortic stenosis with preserved ejection fraction, often seen in elderly hypertensive individuals with small LV size and significant hypertrophy, diagnosis can be complex. It requires ruling out measurement errors and other causes of echocardiographic findings and may involve checking for symptoms, LV hypertrophy, and reduced longitudinal strain. Cardiac computed tomography (CCT) can help by quantifying valve calcification, with certain thresholds suggesting the likelihood of severe aortic stenosis. Conversely, normal-flow, low-gradient aortic stenosis with preserved ejection fraction typically indicates moderate severity of the condition [5].

### 3.3. Exercise Prescription and Competitive Sport Participation

Individuals without symptoms who have mild aortic stenosis can engage in all forms of sports. Asymptomatic patients with moderate AS, normal LV function, and no significant issues during maximal exercise tests may also engage in low-to-moderate intensity sports, if desired. However, athletes with moderate AS showing symptoms, LV dysfunction, or complex arrhythmias should avoid competitive and recreational sports but can maintain general physical activity for cardiovascular health. Those who are asymptomatic but have severe aortic stenosis should avoid all competitive and leisure sports, except for low-intensity skill-based sports. Nonetheless, low-intensity aerobic activities may be recommended for these asymptomatic individuals to enhance functional capacity and overall health [9], with close follow-up to monitor for symptom onset. In cases of bicuspid aortic valve (BAV) with enlarged aortic roots, restrictions apply irrespective of aortic valve disease severity, especially in sports that put extra load on the aorta to reduce the risk of aortic dilatation progression and/or aortic rupture. The 2025 AHA/ACC recommendations are slightly less restrictive, considering lower-intensity endurance and strength sports as potential exceptions in asymptomatic severe AS, although the risks of competitive participation are still considered likely to outweigh the benefits [8]. When aortopathy is not present, the guidelines for physical activity for people with bicuspid aortic valve (BAV) are the same as those for individuals with tricuspid aortic valve dysfunction [4].

## 4. Aortic Valve Regurgitation

### 4.1. Pathophysiology

Aortic regurgitation (AR) is characterized by backflow into the left ventricle (LV), resulting from inadequate diastolic cusp coaptation, due to primary defects of valve leaflets or secondary to aortic dilatation. Chronic AR imposes on the LV an incremental combined increase in volume–pressure load, inducing adaptive eccentric hypertrophy and progressive LV dilatation, with initially preserved ejection fraction. As the AR progresses, the LV myocardial reserve gradually fades, then systolic dysfunction and symptoms subsequently appear. Acute AR (e.g., endocarditis, aortic dissection) is a distinct entity characterized by an abrupt surge in LV afterload pressure and volume with sudden hemodynamic decompensation [5,22].

### 4.2. Aortic Dilatation

Aortic regurgitation (AR) is frequently accompanied or caused by annular and aortic root dilatation (e.g., related to BAV aortopathy, connective tissue disorders, or hypertension). Progressive aortic enlargement worsens leaflet coaptation defects, perpetuating a vicious cycle that further augments the regurgitant volume. Strict surveillance is essential when the aortic diameter exceeds 40 mm or when the growth rate is ≥3 mm/year. Surgical thresholds should align with contemporary ESC recommendations, with individualization based on age, body size (e.g., indexing to body surface area), concomitant valve disease, and genetic risk profile. For associated aortic dilatation, the latest ESC guidelines [10] recommend elective replacement at a diameter of ≥55 mm (Class I, Level B). Earlier intervention at ≥50 mm is suggested for patients with the root phenotype (Class I, Level B) or those presenting with high-risk features (such as a rapid growth rate ≥ 3 mm/year, family history, severe AR, or uncontrolled hypertension—Class IIa, Level C). Furthermore, if aortic valve surgery is already indicated for severe AR, concomitant replacement of the aortic root or ascending aorta should be considered at a threshold of ≥45 mm (Class IIa, Level C).

### 4.3. Diagnosis and Risk Stratification

Echocardiography is the leading diagnostic investigation for AR assessment. An integrated approach is required, combining qualitative data (valve anatomy assessment, holodiastolic flow reversal in the descending aorta, continuous wave Doppler density), semi-quantitative indices (vena contracta width, jet density and pressure half-time), quantitative parameters (effective regurgitant orifice area, regurgitant volume and regurgitant fraction), and LV remodelling metrics. When echocardiographic parameters are controversial or the ultrasound window is insufficient, CMR might offer accurate quantification of regurgitant fraction and LV volumes.

Risk stratification of individuals with AR requires overall clinical and instrumental assessment. In particular, the physician should consider: AR severity, presence of symptoms, LV function and dimensions (LVEF ≤ 55% or LV end-systolic diameter >50 mm indicating risk), aortic diameters and growth rate, exercise test performance (e.g., repolarization abnormalities, blood pressure, arrhythmias), and comorbidities [5]. Symptomatic severe AR and asymptomatic severe AR with LV dilatation/dysfunction require surgical correction [5]. Until correction, competitive sports are contraindicated in both Italian and European recommendations [4,5,13].

### 4.4. Exercise Prescription

Exercise prescription in patients with aortic regurgitation (AR) must balance the undeniable cardiovascular and metabolic benefits of regular physical activity with the hemodynamic burden imposed by the regurgitant volume. According to the 2020 ESC Guidelines on sports cardiology, individuals with mild AR can participate in all recreational sports. Similarly, asymptomatic patients with moderate AR, a non-dilated left ventricle (LV) with preserved ejection fraction (LVEF ≥ 50%), and a normal exercise stress test can safely engage in all recreational activities.

For asymptomatic patients with severe AR presenting with preserved LVEF (≥50%), mild-to-moderate LV dilatation, and a normal stress test, low-to-moderate intensity physical activity may be considered [4,12]. However, these individuals require closer clinical and imaging surveillance (on a 6-monthly basis) to enable the early detection of any decline in LV function. Conversely, if LV dysfunction (LVEF ≤ 50%) or exercise-induced arrhythmias are detected in asymptomatic severe AR, moderate- and high-intensity exercise is strictly contraindicated, and only low-intensity recreational activities are permitted. Finally, in symptomatic patients with severe AR, surgical intervention is indicated; therefore, all training programs should be postponed, although low-intensity aerobic exercise alone is still encouraged to maintain functional capacity and general well-being prior to surgery.

### 4.5. Competitive Sport Participation

The evaluation of competitive sports eligibility in patients with aortic regurgitation (AR) differs slightly between the 2020 ESC guidelines [4] and the 2023 Italian COCIS consensus [12] (Table 4). Both frameworks consider regurgitation severity, left ventricular (LV) remodelling, and aortic dimensions, but the former offers an individualized clinical approach, whereas the latter applies stricter thresholds required for medical certification.

Indeed, according to the ESC guidelines, athletes with mild or moderate AR and preserved LV function (LVEF ≥ 50%) are eligible for all competitive sports, provided their exercise stress test is normal. Even in cases of severe but asymptomatic AR with preserved LVEF and mild-to-moderate LV dilatation, participation in low-to-moderate intensity sports can be considered. Nevertheless, competitive activity is strictly contraindicated if patients develop symptoms, LV systolic dysfunction (LVEF < 50%), or exercise-induced arrhythmias. In contrast, the 2025 AHA/ACC guidelines adopt a more permissive approach to asymptomatic severe AR, considering competitive sport participation reasonable when LV systolic function and diameters are normal or consistent with physiological athletic remodelling, pulmonary hypertension is absent, and exercise tolerance is adequate [8].

The COCIS setting similarly allows full participation for mild AR with normal cardiac dimensions, whereas moderate AR generally restricts athletes to “Group A” sports (low cardiovascular demand), with higher-intensity disciplines permitted only in carefully selected and monitored cases. Furthermore, severe AR represents an absolute contraindication for all competitive sports, regardless of symptom status. Following surgical correction, COCIS may grant eligibility for “Group A” sports to athletes with well-functioning biological prostheses, whereas mechanical prostheses requiring chronic anticoagulation remain an absolute contraindication for any competitive activity.

## 5. Percutaneous and Surgical Aortic Valve Repair and Replacement

### 5.1. Risk Stratification After Tavi and Savr

Transcatheter aortic valve implantation (TAVI) has progressively expanded from patients at prohibitive or high surgical risk to selected intermediate- and low-risk populations, supported by randomized trials showing clinical outcomes comparable to SAVR in appropriately selected patients [6,23,24]. This evolution has substantially broadened the population potentially referred to post-procedural rehabilitation and exercise programs. Current ESC/EACTS guidelines recommend TAVI in patients aged ≥70 years with tricuspid aortic stenosis and suitable anatomy, whereas SAVR remains preferred in patients <70 years at low surgical risk; in patients outside these categories, treatment selection should be individualized by the Heart Team according to clinical characteristics, anatomy, life expectancy, expected valve durability, and patient preference [6].

Despite major advances in device technology and procedural outcomes, differences between TAVI and SAVR remain clinically relevant when planning subsequent physical activity. TAVI is associated with a higher incidence of paravalvular regurgitation and permanent pacemaker implantation, whereas surgical treatment entails greater early procedural invasiveness and a longer initial recovery [24,25]. In younger patients, long-term durability remains an important consideration, and evidence is less robust than in older populations [6,26,27]. Moreover, in patients with BAV—particularly when significant aortopathy coexists—SAVR generally remains the preferred strategy because it allows concomitant treatment of the ascending aorta when indicated [6].

Accordingly, post-intervention risk stratification before exercise prescription should not be based solely on the type of valve procedure. Reassessment should include prosthetic valve function, residual or paravalvular regurgitation, left ventricular function, pulmonary pressures, rhythm and conduction abnormalities, exercise capacity, and the presence of residual aortic disease [6,28]. These factors, together with age, comorbidities, antithrombotic therapy, and the haemodynamic demands of the intended activity, should guide the timing and intensity of rehabilitation and subsequent sports participation.

### 5.2. Exercise Prescription After Tavi

The introduction of TAVI and its rapid evolution as an alternative therapeutic choice to surgical valve replacement has led to the emergence of a “new” target population of valvular heart disease patients requiring tailored cardiac rehabilitation (CR) and exercise training (ET) programs. In the scientific literature, a growing number of observational and randomized studies analysed the effects of CR and ET in patients after TAVI comparing with counterparts undergoing open surgery. Early observational studies evaluated the effect of short-term CR programs on patients after TAVI, showing an improvement in 6-min walking distances and in parameters of physical and cognitive function. The results of these small studies were confirmed in a meta-analysis showing overall improvements in the 6-min walking distance and the Barthel Index as a measure of frailty [27]; however, the small number of included patients and the heterogeneity of the interventions precluded definitive conclusions about the effect of specific types of intervention. A recent observational study evaluated the impact of rehabilitation on mortality 6 months after TAVI in a large cohort of 1017 patients, showing a lower mid-term mortality only in patients participating in CR [28]. In patients after TAVI, combined exercise training (endurance and resistance components) is safe and effective and often results in improved quality of life and exercise capacity. However, TAVI procedures are mainly used in elderly patients with comorbidities that make competitive sports in these individuals almost impossible. The randomized controlled SPORT: TAVI pilot trial showed that ET can be regarded as a safe treatment modality even in very old and frail patients, provided they are still physically able to use strength training machines, at least at low intensities [29]. Even in young subjects undergoing TAVI, the judgment of suitability for competitive sporting activity will be largely conditioned by comorbidities and underlying heart disease and will have to be evaluated on a case-by-case basis in centers with extensive clinical experience.

### 5.3. Exercise Prescription After SAVR

**Figure 1:**
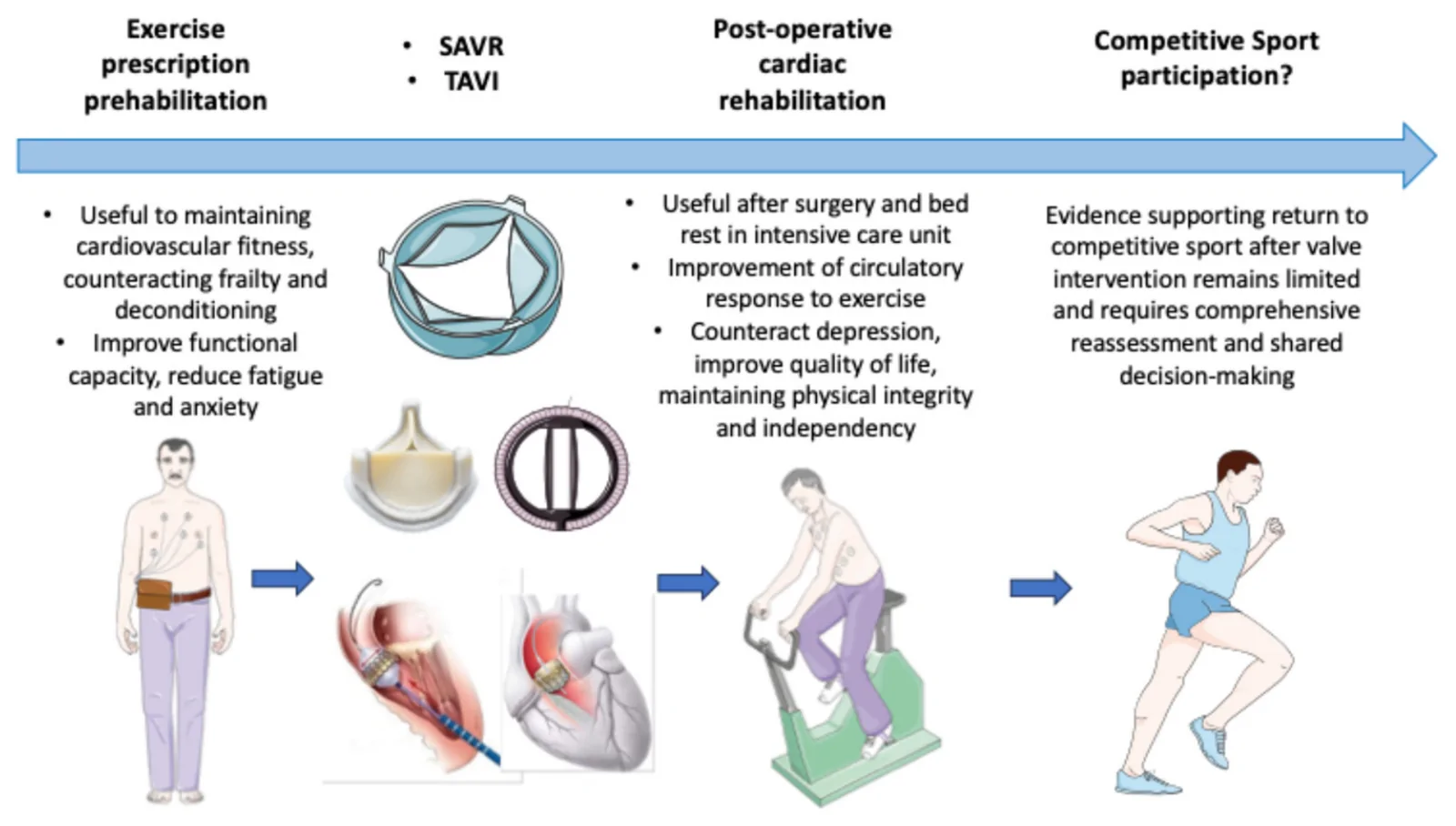
Pre- and post-operative cardiac rehabilitation and exercise training should be individualized. Evidence supporting return to competitive sport after valve intervention remains limited and requires comprehensive reassessment and shared decision-making. SAVR: surgical aortic valve replacement; TAVI: transcatheter aortic valve implantation.

While the benefits of cardiac rehabilitation (CR) after coronary revascularization are widely demonstrated in the scientific literature, only very few studies are available demonstrating the effects of ET in valvular heart disease (VHD) patients following cardiac surgery. The exercise training is particularly advisable after years of physical activity restriction due to the underlying valvular disease and useful after the surgical trauma and the inactivity in the intensive care unit. A key strength of ET after surgical valve replacement is to maximize cardiovascular fitness with improvements in circulatory response to exercise and at the same time to counteract anxiety and depression, improving patient’s quality of life. On the other hand, no standardized protocols, no large randomized controlled trials (RCTs) and limited evidence compared to cardiac rehabilitation in heart failure or after revascularization procedures, represent a clear weakness of ET in heart valve disease. The largest CR trial performed in these patients showed an improvement in cardiovascular fitness by demonstrating an increase in VO_2_ peak but not in the mental health score as evaluated by the SF-36 questionnaire. The current evidence does not allow any reliable conclusions about the effectiveness of CR after valve surgery and the cost-effectiveness for exercise-based CR after surgery has not been fully demonstrated. However, post-operative ET is particularly valuable in young people who aim to return to work and exercise early after surgery and in older individuals to maintain their physical integrity and independence. Figure 1 summarizes these concepts.

### 5.4. Competitive Sports Participation in Patients Undergoing Percutaneous and Surgical Aortic Valve Repair and Replacement

The landscape of competitive sport participation for patients who have undergone percutaneous or surgical aortic valve repair and replacement is evolving, marked by cautious optimism and tailored approaches. Historically, engaging in competitive sports was largely discouraged for these patients due to concerns about the intense physical strain and its potential impact on the repaired or replaced valve. However, recent advancements in surgical and percutaneous valve repair techniques, such as Transcatheter Aortic Valve Replacement (TAVR), have opened new possibilities for these individuals [28]. The decision to engage in competitive sports post-valve repair or replacement should be individualized, taking into account the patient’s overall health, type of sport, and the specific details of their valve intervention [30]. Multidisciplinary teams, including cardiologists, surgeons, and sports medicine specialists, play a crucial role in guiding these patients, ensuring that their return to competitive sports is safe and aligned with their cardiovascular health goals.

Current evidence suggests that with appropriate risk assessment [31], some forms of competitive sports might be viable for certain post-procedure patients, especially for skill disciplines or low-intensity sport [9,32]. However, evidence specifically addressing return to competitive sport after valve intervention remains limited, and the long-term safety of intensive exercise in this population is not well established [4,9]. Current recommendations therefore rely largely on expert consensus, individualized risk stratification, and shared decision-making [4,8]. Importantly, evidence supporting cardiac rehabilitation and structured exercise training after valve intervention should not be interpreted as elements supporting the safety of unrestricted competitive sport. A key factor in this decision-making process is the type of valve repair or replacement performed. Mechanical valve replacements, for instance, require lifelong anticoagulation, which may limit participation in contact sports due to bleeding risks [9]. This contrasts with the more individualized AHA/ACC approach, in which restriction is driven primarily by the bleeding risk associated with anticoagulation and the collision or impact characteristics of the specific sport, rather than by the mechanical prosthesis itself [8]. Conversely, patients with bioprosthetic valves or successful percutaneous interventions may face fewer restrictions, provided their cardiac function is adequately preserved and closely monitored [10]. According to 2021 European guidelines on valvular heart disease [10], it is recommended that athletes who have had valve replacements have the same exercise limitations as asymptomatic athletes with moderate native valve disease provided ventricular function is preserved and pulmonary artery pressure is within normal limits. Despite the limited evidence supporting return to competitive sport after valve intervention, the 2025 AHA/ACC statement is more permissive than previous European recommendations [4,8], considering competitive sport participation reasonable in athletes with bioprosthetic valves and normal LV systolic function after appropriate post-operative recovery and functional reassessment [8].

## 6. Discussion and Future Perspectives

The intersection of aortic valve disease (AVD), associated aortopathies, and sports cardiology is undergoing a profound paradigm shift. Historically, the theoretical fear of exercise-induced adverse events—such as accelerated valvular deterioration or acute aortic dissection—led to highly restrictive recommendations [32]. However, a modern clinical perspective seeks to prevent a sedentary lifestyle, which remains a primary modifiable cardiovascular risk factor. Consequently, the contemporary clinical focus has evolved from blanket prohibition to exploring how exercise can be safely prescribed to maximize cardiometabolic health in these patients [4]. Although European and North American recommendations are broadly concordant, the 2025 AHA/ACC guidance suggests a more permissive shared decision-making scheme in selected clinical scenarios, as previously highlighted [8].

A critical challenge in this field is distinguishing physiological adaptation from early pathology. Systematic screenings of competitive athletes have shown that highly trained individuals exhibit slightly larger aortic root diameters compared to sedentary controls [3,4]. However, this difference is often blunted when indexed for body surface area (BSA), suggesting that the “athlete’s aorta” is primarily a physiological adaptation driven by increased stroke volume and body size, rather than a pathological remodelling process [20,33]. Although acute events during sports are extremely rare, they are mostly associated with heavy isometric disciplines that provoke abrupt and massive blood pressure surges without the compensatory drop in peripheral resistance seen in dynamic exercise [20,34,35].

Recent pathophysiological insights emphasize that BAV-associated aortopathy is largely driven by altered transvalvular flow, generating asymmetrical wall shear stress, which disrupts the endothelial nitric oxide pathway and elevates oxidative stress within the aortic media [13]. In these patients, concern has traditionally centered on strenuous resistance training [36,37]—particularly heavy power disciplines—which induces massive pressure overload without the compensatory drop in peripheral vascular resistance seen in purely dynamic exercise [20], and which could exacerbate mechanical wall stress and serve as a trigger for acute aortic syndromes [4]. Interestingly, other evidence challenges the assumption that all forms of strenuous exercise are detrimental to the diseased aorta. Longitudinal observational studies tracking competitive athletes with BAV demonstrated that their annual rate of aortic expansion does not significantly differ from the growth rates observed in the general, non-athletic BAV population [38,39].

Furthermore, data from translational research in murine models of Marfan syndrome (MFS) suggest a paradoxical protective effect of aerobic activity. In these models, mild-to-moderate dynamic exercise reduced the rate of aortic root dilatation, blocked elastin fiber fragmentation, and improved the overall mechanical strength of the aortic wall [40,41].

Despite these encouraging physiological insights, significant knowledge gaps remain. The major limitation in contemporary sports cardiology is the reliance on cross-sectional registries and expert consensus, given the conspicuous absence of large, prospective randomized controlled trials (RCTs) assessing the longitudinal impact of specific exercise intensities on thoracic aortopathies, similar to the existing RCTs in abdominal aortic aneurysms that successfully demonstrated no adverse effects of exercise on aneurysm growth [42]. Future research must prioritize such prospective cohorts to define precise safety thresholds.

Additionally, the integration of advanced multimodality imaging could represent a revolution. Innovations such as 4D-flow cardiovascular magnetic resonance (CMR) allow for the in vivo mapping of blood flow, serving as a highly promising non-invasive predictor of individual responses to physical stress [12,43]. In addition, insights from translational medicine and cardiology will provide novel biomarkers to guide therapy and follow-up [44]. Combined with deeper genetic profiling and routine cardiopulmonary exercise testing (CPET) [4], these tools will empower clinicians to safely prescribe highly personalized regimens as a part of a “precision sports cardiology” program. Last but not least, it is mandatory to exclude additional risk factors related to the use of doping agents. Indeed, anabolic-androgenic steroids and other performance-enhancing substances may increase systemic blood pressure and promote arrhythmias [1,2], potentially increasing cardiovascular risk and haemodynamic stress on the aortic wall in athletes with underlying valvular disease or aortopathy [45].

## 7. Conclusions

Prescribing exercise for patients with aortic valve disease requires balancing cardiometabolic benefits against potential hemodynamic risks. Contemporary sports cardiology has shifted from absolute restrictions toward individualized prescriptions. Current guidelines agree that asymptomatic individuals with mild-to-moderate AVD, preserved ventricular function, and normal aortic dimensions can safely engage in competitive sports. Conversely, severe valvulopathy, LV dysfunction, or significant aortopathy necessitates sports restriction and surgical evaluation. Nevertheless, rigorous imaging surveillance, exercise stress testing, and shared decision-making are essential to safely optimize athletic participation in this population.

## Figures and Tables

**Table 1:** Clinical evaluation algorithm of patients with aortic valve disease. CPET: cardiopulmonary exercise test; EST: exercise stress test.

Clinical Evaluation Algorithm of Patients with Aortic Valve Disease
**1.** Family and clinical history
**2.** Physical examination, including features of genetic/familial disease
**3.** 12-lead resting ECG
**4.** Echocardiography and/or other advanced imaging techniques
**5.** CPET (if not available, EST)
**6.** 24-h ambulatory ECG monitoring including a training session
**7.** Ambulatory Blood Pressure Monitoring (ABPM)
**8.** Recommendation on type, intensity, duration of exercise; Clearance for competitive sport
**9.** Follow-up

**Table 2:** Bicuspid aortic valve: European Society of Cardiology (ESC, 2020), American Heart Association/American College of Cardiology (AHA/ACC, 2025), Italian recommendations for competitive sports participation (COCIS, 2023), and Italian Society of Sports Cardiology/Italian Federation of Sports Medicine (SIC Sport/FMSI) recommendations for exercise prescription.

BAV	ESC 2020—Competitive Sport	COCIS 2023—Competitive Sport	AHA/ACC 2025—Competitive Sport	SIC Sport/FMSI—Exercise Prescription	Surveillance/Notes
Normally functioning BAV; normal aorta (<40 mm); normotensive	All competitive sports allowed	Class I—eligible; all sports if normotensive and normal testing	Competitive sports participation allowed	All types of exercise, intensities and frequencies	Echo/CMR-CTA every 2–3 years
Mild AS/AR; normal aorta (<40 mm)	Competitive sport generally permitted if normal exercise testing	Class II—conditional; individualized assessment	Competitive sports participation permitted	Moderate-to-high-intensity exercise, individualized	Periodic clinical/echo assessment and exercise testing
Aortic 40–44 mm without additional risk factors *	Low–moderate dynamic and low-static sports; avoid high-static/power activities	Class II—conditional; low-static and low-dynamic disciplines after individualized assessment	Competitive sport may be considered with SDM, accounting for age, sex, body size and additional aortic risk factors *	Individualized exercise; avoid marked BP surges	Annual imaging and exercise BP monitoring
Aorta ≥ 45 mm	Competitive sport generally contraindicated	Class III—not eligible for competitive sport	Risks likely outweigh benefits; competitive sport may still be considered in selected athletes after SDM and expert consultation	Individualized low-intensity exercise	Close longitudinal surveillance; assess surgical indications
Moderate–severe AS/AR, irrespective of aortic dimensions	Eligibility determined by the specific valve lesion; severe disease generally restricts competitive sport	Class III—not eligible	Follow lesion-specific recommendations: eligibility depends on lesion severity, symptoms, LV function/remodelling, pulmonary pressure and exercise response (see Table 4)	Individualized according to valve severity	Clinical/imaging follow-up and exercise testing
Syndromic or heritable thoracic aortic disease (HTAD) ^†^/additional high-risk factors *	Generally restrictive; individualized according to underlying condition and aortic dimensions	Usually Class III; exceptional individualized low-risk cases	Individualized according to genotype/phenotype, aortic dimensions and additional risk factors *; high-intensity strength exercise is of particular concern	Individualized exercise prescription	Specialist aortic/genetic assessment and close imaging surveillance
Postsurgical repair of BAV-associated aortopathy, with stable imaging	Return may be considered after recovery; sport selection individualized	Specialist certification; initially low-static/low-dynamic disciplines	Competitive sport is reasonable after complete sternal healing, with SDM, expert consultation and longitudinal surveillance	Individualized exercise prescription	Prosthesis/graft and residual-aorta surveillance

Notes: * Additional risk factors for aortic dissection: family history of aortic dissection, rapid aortic growth (≥3 mm/year), untreated substantial aortic coarctation, or features suggesting an underlying HTAD. ^†^ HTAD: syndromic and non-syndromic heritable thoracic aortic diseases. Sports-related risk should be individualized according to the underlying diagnosis/genotype, aortic dimensions, body size, family history and other disease-specific risk features rather than considering HTAD as a homogeneous entity. Abbreviations: AHA/ACC, American Heart Association/American College of Cardiology; AR, aortic regurgitation; AS, aortic stenosis; BAV, bicuspid aortic valve; BP, blood pressure; CMR, cardiac magnetic resonance; COCIS, Italian Sports Cardiology Guidelines for competitive sport practice; CTA, computed tomography angiography; ESC, European Society of Cardiology; HTAD, heritable thoracic aortic disease; LV, left ventricular; SDM, shared decision-making; SIC Sport/FMSI, Italian Society of Sports Cardiology/Italian Federation of Sports Medicine.

**Table 3:** Echocardiographic assessment of the severity of aortic stenosis.

Stenosis Degree	Vmax (m/s)	Mean Gradient (mmHg)	Valve Opening Area (cm^2^)	Valve Opening Area Index (cm^2^/m^2^)	Velocity Ratio
Mild	2.6–2.9	<20	>1.5	>0.85	>0.50
Moderate	3.0–4.0	20–40	1.0–1.5	0.60–0.85	0.25–0.50
Severe	≥4.0	≥40	<1.0	<0.6	<0.25

**Table 4:** Aortic regurgitation and aortic stenosis: European Society of Cardiology (ESC, 2020) vs. Italian recommendations for competitive sports participation (COCIS, 2023) and Italian Society of Sports Cardiology/Italian Federation of Sports Medicine (SIC Sport/FMSI) exercise prescription in patients with cardiovascular diseases.

Aortic Regurgitation/Aortic Stenosis	ESC 2020—Competitive Sport	COCIS 2023—Competitive Sport	AHA/ACC 2025—Competitive Sport	SIC Sport/FMSI—Exercise Prescription	Surveillance/Notes
Mild AS or AR; normal LV function; normal aorta	All competitive sports generally allowed with normal exercise response	Class I—eligible if normotensive with normal testing	Competitive sports participation allowed	All types of exercise, intensities and frequencies	Periodic echo; BP control
Moderate AS; asymptomatic; preserved LV function	Competitive sport may be considered with normal functional capacity, exercise BP response and absence of complex arrhythmias on maximal exercise testing	Class II—conditional; generally limited to lower-demand disciplines	Competitive sports participation is reasonable with SDM when maximal exercise testing is normal	Moderate-to-high-intensity exercise may be considered individually	Annual clinical/echo assessment; maximal exercise testing
Severe AS; asymptomatic	Competitive sport generally contraindicated; low-intensity skill sports may be considered	Class III—not eligible	Risks likely outweigh benefits, except for lower-intensity endurance and strength sports; valve intervention should be considered with SDM	Active lifestyle; low-intensity activity	Close follow-up and evaluation for intervention
Symptomatic severe AS	Competitive sport contraindicated	Class III—not eligible until intervention and clinical stability	Competitive sport should not be performed; valve intervention should be considered	Active lifestyle only, according to clinical status	Valve intervention indicated/evaluated
Moderate AR; asymptomatic; preserved LV function	All competitive sports may be considered with preserved LV function and normal exercise testing	Class II—conditional; generally restricted	Competitive sports participation is reasonable when preserved LV systolic function, normal ventricular dimensions or consistent with physiological athletic remodelling, PH is absent and exercise tolerance is normal	Moderate-to-high-intensity exercise may be considered	Annual clinical/echo review; exercise testing
Severe AR; asymptomatic; preserved LV function	Low-to-moderate-intensity competitive sport may be considered with preserved LV function, mild-to-moderate LV dilatation and normal exercise testing	Class III—not eligible for competitive sport	Competitive sports participation is reasonable when preserved LV systolic function, normal ventricular dimensions or consistent with physiological athletic remodelling, PH is absent and exercise tolerance is normal	Individualized exercise prescription	Close imaging surveillance
Severe AR with LV systolic dysfunction/pathological LV dilatation, PH or clinically relevant exercise-induced ventricular arrhythmias	Competitive sport contraindicated	Class III—not eligible	Risks likely outweigh benefits; further clinical evaluation and consideration of valve intervention are warranted	Low-intensity exercise according to clinical status	Surgical evaluation and close follow-up
Post-valve surgery; bioprosthetic valve with normal LV systolic function	Conservative individualized approach; exercise limitations broadly comparable with asymptomatic moderate native VHD	Conditional eligibility; predominantly lower-demand sports	Competitive sports participation is reasonable after appropriate post-operative recovery and reassessment *	Individualized exercise prescription	Prosthetic valve assessment; maximal-effort exercise testing
Mechanical prosthetic valve requiring indefinite anticoagulation	Participation primarily limited by anticoagulation and sport-specific trauma risk	Competitive sport considered contraindicated	Participation depends primarily on anticoagulation-related bleeding risk and the collision/impact characteristics of the sport	Individualized exercise prescription	Anticoagulation and sport-specific bleeding-risk assessment

Notes: * Postsurgical evaluation should include a maximal-effort exercise stress test. Resumption of competitive sport should be individualized after complete sternal healing and participation in postsurgical exercise rehabilitation. Competitive sports participation is considered reasonable in athletes with bioprosthetic valves and normal LV systolic function. Abbreviations: AHA/ACC, American Heart Association/American College of Cardiology; AR, aortic regurgitation; AS, aortic stenosis; BP, blood pressure; COCIS, Italian Sports Cardiology Guidelines for competitive sport practice; ESC, European Society of Cardiology; LV, left ventricular; LVEF, left ventricular ejection fraction; PH, pulmonary hypertension; SDM, shared decision-making; SIC Sport/FMSI, Italian Society of Sports Cardiology/Italian Federation of Sports Medicine; VHD, valvular heart disease.

## Data Availability

No new data were created or analyzed in this study. Data sharing is not applicable to this article.

## Abbreviations

The following abbreviations are used in this manuscript:

AR	Aortic regurgitation
AS	Aortic stenosis
AVD	Aortic valve disease
BAV	Bicuspid aortic valve
BP	Blood pressure
CMR	Cardiac magnetic resonance
COCIS	Italian Sports Cardiology Guidelines for competitive sport practice
CPET	Cardiopulmonary exercise test
CTA	Computed tomography angiography
ECG	Electrocardiogram
EST	Exercise stress test ECG
ESC	European Society of Cardiology
FMSI	Italian Federation for Sports Medicine
LV	Left ventricular
LVEF	Left ventricular ejection fraction
MDT	Multidisciplinary team
SAVR	Surgical aortic valve replacement
TAV	Tricuspid aortic valve
TAVI	Transcatheter aortic valve implantation
TEE	Trans-oesophageal echocardiography
TTE	Transthoracic echocardiography
